# Relationships between Bacterial Community Composition, Functional Trait Composition and Functioning Are Context Dependent – but What Is the Context?

**DOI:** 10.1371/journal.pone.0112409

**Published:** 2014-11-07

**Authors:** Ina Severin, Eva S. Lindström, Örjan Östman

**Affiliations:** Department of Ecology and Genetics/Limnology, Uppsala University, Uppsala, Sweden; Argonne National Laboratory, United States of America

## Abstract

Bacterial communities are immensely diverse and drive many fundamental ecosystem processes. However, the role of bacterial community composition (BCC) for functioning is still unclear. Here we evaluate the relative importance of BCC (from 454-sequencing), functional traits (from Biolog Ecoplates) and environmental conditions for per cell biomass production (BPC; ^3^H-leucine incorporation) in six data sets of natural freshwater bacterial communities. BCC explained significant variation of BPC in all six data sets and most variation in four. BCC measures based on 16S rRNA (active bacteria) did not consistently explain more variation in BPC than measures based on the 16S rRNA-gene (total community), and adding phylogenetic information did not, in general, increase the explanatory power of BCC. In contrast to our hypothesis, the importance of BCC for BPC was not related to the anticipated dispersal rates in and out of communities. Functional traits, most notably the ability to use cyclic and aromatic compounds, as well as local environmental conditions, i.e. stoichiometric relationships of nutrients, explained some variation in all six data sets. In general there were weak associations between variation in BCC and variation in the functional traits contributing to productivity. This indicates that additional traits may be important for productivity as well. By comparing several data sets obtained in a similar way we conclude that no single measure of BCC was obviously better than another in explaining BPC. We identified some key functional traits for productivity, but although there was a coupling between BCC, functional traits and productivity, the strength of the coupling seems context dependent. However, the exact context is still unresolved.

## Introduction

Lakes and reservoirs are central compartments in the global carbon cycle [1], through the processing of organic matter by microorganisms, making resources available for higher trophic levels of the grazer chain [2] or contributing to the outgassing of carbon dioxide to the atmosphere [3], [4]. It is well known that local environmental conditions such as temperature and availability of nutrients are related to bacterial production [5]; the question is, however, if also bacterial community composition (BCC) plays a role. Results obtained so far are rather inconclusive, both in field [6], [7] and experimental studies [8]–[12].

Disparate results may rise from methodological differences, for instance due to the fact that in natural bacterial communities a substantial proportion of the cells may be inactive or dormant [13], and BCC measures including such cells may obscure BCC- function relationships. Further, results could be affected by the choice of method to classify operational taxonomic units (OTUs), i.e. whether sequence similarities alone or also phylogenetic distances between taxa [14], [15] are taken into account. Using a phylogenetic diversity measure may result in a tighter observed coupling between BCC and functioning if closer related taxa are functionally more similar. However, carbon processing traits tend to be dispersed in the 16S rRNA phylogeny [16], i.e. they are shared in phylogenetically shallow clusters all across the bacterial realm. Therefore, any BCC measure may have a limited explanatory power for productivity compared to measurements of key functional traits. For instance, in a previous study [12], we showed that the community productivity of heterotrophic bacteria is dependent on the community's ability to use certain carbon substrates in habitats where these substrates are abundant. Thus, functional trait composition may have a better explanatory power for functioning than BCC.

In addition to the methodological issues, the strength of BCC-functioning relationships can differ due to ecological reasons, depending for instance on the proportion of generalists and specialists in a community, since a great proportion of generalists should result in weaker BCC-functioning relationships [17]. The degree of generalism may in turn depend on community assembly mechanism and environmental heterogeneity. Dispersal may, for example, favor resource generalists [18], and BCC and functioning should therefore be uncoupled if the dispersal rate is high. In contrast, large differences in environmental conditions and low (but not limiting) dispersal among communities could favor resource specialist taxa via species sorting processes and, thus, stronger BCC-functioning relationships are expected [7], [19].

For a better understanding of the importance of BCC for community functioning we need a conceptual framework and systematic studies including both methodological awareness and ecological considerations. Here we used six different data sets of aquatic bacteria, from 7–15 communities each, to explore the relative importance of the local environment, carbon processing trait composition and BCC for the productivity of bacterial communities depending on dispersal rates and environmental heterogeneity among communities. Two of the data sets originate from lake sediment communities, two from the epilimnion communities of lakes and two from stream communities. The ecosystem function of interest in this study is bulk bacterial community production (leucine incorporation) and the different functional traits under consideration are the communities' ability to use different carbon substrates (assessed from Biolog-plates). Bacterial taxonomic community composition (BCCt) and phylogenetic community composition (BCCp) were determined by high-throughput DNA sequencing, hypothesizing that BCCp would explain more variation in productivity than BCCt. Further, our BCC measures were based on the 16S rRNA (rBCC) as well as the 16S rRNA gene (dBCC). Assuming that rBCC would better reflect the active part of the community [20] we hypothesize that rBCC will be more closely linked to productivity than measures based on the total community (dBCC). Finally, we hypothesize that BCC (incorporating all aspects of BCC) would explain less variation in productivity with increasing dispersal rates and/or lower environmental heterogeneity among communities in a data set because these conditions are assumed to favor habitat generalism. In contrast, functional trait composition would not be affected by the degree of generalism and should therefore explain relatively more of the variation in productivity with increasing dispersal rates and/or higher environmental heterogeneity.

## Material and Methods

All abbreviations are given as an overview in Table S1.

### Ethics Statement

No permits were required to sample any of the water bodies in this study. The authors also confirm that the sampling did not affect endangered or protected species.

### Sampling

Three freshwater systems in two geographic regions in Sweden were sampled in summer and autumn 2010; pelagic lake water (epilimnion), lake sediments (upper 1 cm) and stream waters. One of the lake systems is situated in the province of Jämtland (approximately 63°N and 13°E) where all the 14 sampled lakes are connected to the river Indalsälven either by an inlet and/or an outlet. Samples were obtained in June from all pelagic lake waters (Jw) and 7 sediments (Js) (due to harsh weather conditions the sediments in the remaining seven lakes could not be sampled). The other lake-system is situated in Uppland (approximately 60°N and 17°E), where water (Uw) and sediments (Us) were sampled in 15 hydrologically unconnected lakes in June. Stream samples were obtained from 15 sites in River Fibyån, Uppland, in July (S I) and September (S II). We assumed that 1) dispersal of bacterial cells among communities was lowest in sediments since it requires both sediment resuspension within lakes and dispersal among lakes; 2) dispersal rates among stream water communities was highest due to shorter water retention time in streams in relation to lakes, but that dispersal was greater in S II compared to S I since water levels indicated a higher water flow in September; 3) among the lakes, dispersal was higher among the Jämtland lakes since they are all part of the same river system while the Uppland lakes were not. Based on these assumptions the data sets were ranked from 1–6 where 6 denotes the highest dispersal rate (S II) and 1 the lowest (Us).

### Environmental data

Non-purgeable total organic carbon (hereafter termed TC) in water samples was determined by measuring organic carbon after acidification with HCl (TOC-5000, Shimadzu, Kyoto, Japan). Total nitrogen (TN) in water samples was measured spectrophotometrically (Hitachi U-2000, Hitachi, Ltd., Tokyo, Japan) as nitrate after oxidation at high temperature. Total phosphorus (TP) in water and sediment samples was also measured spectrophotometrically after oxidative hydrolysis of organically bound phosphorus. Total carbon (TC) and total nitrogen (TN) in sediment samples was determined in freeze-dried and ash-free sediments by combustion with oxygen (elemental combustion system, Costech Analytical Technologies, Inc., Valencia, CA, USA).

The Jämtland lakes are generally more oligotrophic with low levels of total organic carbon compared to all Uppland sites (Table 1). However, the environmental variability, measured as coefficient of variation (CV) among sites, was not consistently higher in any data set but differed between environmental variables (Table 1).

**Table 1:** Dispersal, environmental conditions and heterogeneity (measured as coefficient of variation, CV) in the data sets.

Data set	Dispersal category	TC	TN	TP	TN/TC	TP/TC	CV TC	CV TN	CV TP
Jw	1	4	0.1	15	0	4.5	43.5	60.5	60.6
Uw	2	21.7	1.1	46.6	0.03	4.46	32.2	29.6	73.3
Js	3	4.3	0.4	0.1	0.05	2.31	54.8	45.3	38.9
Us	4	15	1.1	0.1	0.1	0.02	44.6	39.1	23.8
S I	5	29.4	1.9	93.9	0.08	0.01	44.5	53.2	78.3
S II	6	31.8	2	103.9	0.07	4.08	50.1	33.3	106.2

TC: total organic carbon [mg l^−1^ for water and % dry weight for sediments], TN: total nitrogen [mg l^−1^ for water and % dry weight for sediments], TP: total phosphorus [µg l^−1^ for water and % dry weight for sediments].

### Bacterial abundance

Cell abundances were determined flow-cytometrically [21] (CyFlow space, Partec GmbH, Münster, Germany) for water samples and microscopically (Nikon Eclipse E600 fluorescence microscope, Nikon Corporation, Tokyo, Japan) for sediment samples. Following the protocol by [21] water samples were fixed with filtered formaldehyde (3.7% final concentration) and stored at 4°C for a maximum of two days. The cells were stained with SYTO 13 (Invitrogen, Life Technologies Ltd, Paisley, UK). The sediment samples were diluted 10× with filtered lake water (0.2 µm filters, Supor-200 Membrane Disc Filters, 47 mm; Pall Corporation, East Hills, NY, USA) and fixed with filtered formaldehyde (3.7% final concentration). This sediment slurry was then diluted 500× with an 50/50 mix of tap water and deionized water and sonicated at 100 W for 1 min on ice. After settlement of the particles, the cells in the supernatant were stained with DAPI (4′,6-Diamidino-2-Phenylindole, Dihydrochloride, Invitrogen, Life Technologies Ltd, Paisley, UK) for 15 min and filtered onto 0.2 µm black polycarbonate filters (Sorbent AB, Västra Frölunda, Sweden). At least 10 fields with at least 200 cells in total were counted for each filter.

### Bacterial production

The incorporation of ^3^H-labelled leucine into bacterial protein in water samples was determined using the modified method from [22]. In short, for each sample two parallels and a blank (immediate addition of a final concentration of 5% TCA) were incubated in a final concentration of 100 nM ^3^H-leucine for one hour in the dark at close to ambient temperatures (the same temperature for all samples within a data set). The incubation was stopped by adding a final concentration of 5% TCA to the water samples. After washing with 5% TCA and 80% Ethanol, 0.5 ml of the scintillation cocktail (Optiphase Hisafe 2, PerkinElmer, Inc., Waltham, MA, USA) were added and the samples were kept for at least 24 hours before measurement of the incorporated ^3^H-leucine (Packard Tri-Carb 2100TR Liquid Scintillation Analyzer, GMI, Inc, Ramsey, MN, USA). For sediment samples, homogenized sediment was diluted 1000× with sterile-filtered (0.2 µm-filter) lake water from the same location. This sediment slurry was incubated with a final concentration of 10 µM ^3^H-leucine for one hour. Samples and blanks were then treated as described for the water samples.

Average per cell productivity (BPC) was inferred as bacterial production (BP)/bacterial abundance (BA). This was done in order to obtain a measure of bulk community productivity independent of abundance.

### Functional trait composition

Functional trait composition of communities was assessed as the capacity of the communities to use different carbon sources. 150 µl of either water or a 1000× dilution of the sediment were incubated on Biolog EcoPlates (Biolog, Inc., Hayward, CA, USA). These 96-well plates contain 3 sets of 31 carbon substrates and a water blank. The use of these substrates was followed by absorbance measurement of the colorless tetrazolium dye which is reduced to a violet formazan during oxidation of the substrates by bacterial metabolism. Changes in color development were measured using a microplate reader (TECAN ULTRA 384, Tecan Group Ltd., Männedorf, Switzerland) at 595 nm. Immediately after inoculation, the zero time-point was measured and measurements were repeated daily. The color development was followed until the maximum color development was reached (no further increase in absorbance). The overall color development of each plate was expressed as average well color development (AWCD, [23]) and the absorbance profiles corresponding to the time at which the AWCD was 1.5 AWCD were used. In the analysis substrates were grouped according to their molecular structure into polymers (tween 40 and 80, α-cyclodextrin and glycogen), aromatic compounds (2-hydroxy benzoic acid, 4-hydroxy benzoic acid, L-phenylalanine, phenyletylamine), non-aromatic amino acids (L-arginine, L-asparagine, L-serine, L-threonine, glycyl L-glutamic acid), cyclic compounds other than aromatics (D-cellobiose, α-D-lactose, β-methyl-D-glucoside, D-xylose, N-acetyl-D-glucosamine, glucose-1-phosphate, D-galactonic acid γ-lactone, D-galaturonic acid), and simpler compounds (the rest). The average AWCD-normalized absorbance scores of the substrates in each group were calculated for the PLS analyses (see below).

### Nucleic acid extraction

100 ml of the lake and stream water were filtered onto a 0.2 µm filter (Supor-200 Membrane Disc Filters, 47 mm; Pall Corporation, East Hills, NY, USA). The filters were stored in liquid nitrogen in the field and later at −80°C until further processing. Approximately 0.5 ml of the undiluted sediment was frozen directly. Nucleic acids (DNA and RNA) were extracted using the Easy-DNA kit from Invitrogen (Life Technologies Ltd, Paisley, UK) according to protocol #3. For the extraction of nucleic acids form the filters, glass beads (0.1 mm zirconia/silica, BioSpec Products, Inc., Bartlesville, OK, USA) were added at the beginning of the extraction procedure. The tubes were then shaken in a vortex for 15 min to break filters and cells. No such step was included for the sediment samples. Extracts were quality-checked on a 1% agarose gel. After completion of the protocol, half of the nucleic acid extract was subjected to DNase treatment (DNase I, Invitrogen, Life Technologies Ltd, Paisley, UK) and reverse transcription (RevertAid HMinus First Strand cDNA Synthesis kit, Fermentas Sweden AB, Helsingborg, Sweden) according to the manufacturers' instructions using random hexamer primers. The resulting cDNA as well as the DNA extracts were stored at −80°C until further processing.

### PCR amplification and Template Preparation

The bacterial hypervariable regions V3 and V4 of the 16S rRNA (cDNA) as well as its gene (DNA) were PCR amplified using forward primer 341 5′- CCTACGGGNGGCWGCAG-3′ and individually bar-coded reverse primers 805 5′- GACTACHVGGGTATCTAATCC-3′ [24]. Each 20 µL PCR reaction contained 0.4 U Phusion high-fidelity DNA polymerase (Finnzymes, Espoo, Finland), 1× Phusion HF reaction buffer (Finnzymes), 200 µM of each dNTP (Life Technologies Ltd, Paisley, UK), 250 nM of each primer (Eurofins MWG, Ebersberg, Germany), 0.4 mg mL^−1^ BSA (New England Biolabs, Ipswich, UK) and 5–10 ng of extracted nucleic acid. Thermocycling was conducted with an initial denaturation step at 98°C for 30 sec, followed by 25 cycles of denaturation at 98°C for 10 sec, annealing at 50°C for 30 sec and extension at 72°C for 30 sec, and finalised with a 7-min extension step at 72°C. Three to four technical replicates were run per sample, pooled after PCR amplification and quality-checked on a 1% agarose gel. Purification was carried out using the AMPure XP purification kit (Beckman Coulter Inc., Brea, CA, USA). Nucleic acid yields were then checked on a fluorescence microplate reader (Ultra 384; Tecan Group Ltd., Männedorf, Switzerland) applying the Quant-iT PicoGreen dsDNA quantification kit (Invitrogen, Life Technologies Ltd, Paisley, UK). Finally, PCR amplicons were combined equimolarly, i.e. in equal proportions, to obtain a similar number of 454 pyrosequencing reads per sample.

### 454 Pyrosequencing

The final, pooled amplicon was 454 pyrosequenced with a 454 GS FLX system (454 Life Sciences) at the Norwegian High-Throughput Sequencing Centre, University of Oslo (NSC; Oslo, Norway; http://www.sequencing.uio.no), using Titanium chemistry. Sequences were, prior to analyses, quality-checked and truncated to 400 bases. Each data set was individually processed with AmpliconNoise to reduce the number of PCR and 454 sequencing artifacts and chimeras [25]. 454 pyrosequencing reads have been deposited in the National Center for Biotechnology Information Sequence Read Archive (NCBI-SRA) under accession number SRP016145. For further analysis, singletons as well as sequences belonging to *Archaea* and chloroplasts were removed from the data set. Operational taxonomic units (OTUs) were defined using complete linkage clustering at a level of 99% sequence identity. Computations were performed on resources provided by SNIC through the Uppsala Multidisciplinary Center for Advanced Computational Science (UPPMAX). Taxonomic affiliation (phylum-level) of OTUs was determined by aligning representative sequences to the Greengenes imputed core reference alignment [26] (http://greengenes.lbl.gov) using PyNAST [27] in Qiime [28].

After processing an average of 1450 (median  = 774) and 450 (median  = 347) reads per sample of the total community (DNA) and active bacterial community (cDNA), respectively, were obtained. Because of the risk of large sampling errors with few numbers of reads per sample we omitted samples with <200 reads (1 sample in S I and 4 samples in S II for the total commuinity (DNA), and 1 sample in Js, 4 samples in Us and 2 each in the stream samples for the active community (cDNA)). The range in number of reads between cDNA samples was 200–2445 and for DNA 200–4719.

### Phylogenetic tree

Due to the limited length of the sequenced region (approx. 400 bp) and the large amount of different taxa (over 30 000) we could only construct a robust phylogenetic tree for a subset of the total community. Otherwise the phylogeny would be a random tree without sufficient node support. Therefore we constructed phylogenetic trees for the most abundant and, hence, likely functionally most important taxa. For the 142 taxa of the cDNA data set and the 153 taxa of the DNA data set that had an average relative abundance of 0.1% across all samples or 0.3% in a single data set (18% and 25% of all reads in total and active community, respectively) we constructed 10 000 single phylogenetic trees and the consensus tree from the 16S RNA sequence using a generalized time reversible (GTR) evolutionary model with gamma-distributed rate variation across variable sites in mrBayes 3.2 [29]. The branch length prior was set to a uniform clock. The standard deviations of splits after 10 000 000 generations was <0.005 indicating most nodes were well supported. A tree was sampled every 1000th time step and the last 5 000 trees from the two runs were saved and used for calculating the consensus trees. From the consensus trees we caluculated phylogentic similarities of communities using the Phylogenetic Community Dissimilarity [30] in the picante-package for R. It calculates the pairwise phylogenetic distance among nonshared taxa between two communities, i.e this measure is based only on the occurrence of different taxa and their phylogenetic distance, not the relative abundance of taxa.

### Bacterial community composition

We calculated four different estimates of bacterial community composition for each of the six data sets, i.e. all BCC measures were only calculated for communities within a data set and do not account for between data set compositional differences (which was much larger than within data set differences). For each DNA and cDNA data set we calculated one measure of taxonomic composition without accounting for phylogenetic distance between taxa (dBCCt and rBCCt for total and active community, respectively), and one measure of phylogenetic community composition accounting for phylogenetic distances (dBCCp and rBCCp for total and active community respectively). To extract one measure of rBCCt and dBCCt for each community we used the site scores from the first axis of a Principal Coordinate Analysis (PCoA) of the Morisita-Horn distance matrix (see ‘statistical analyses’ below) for each data set. In this case the first axis explained 12–42% of the variation in rBCCt and 33–70% in dBCCt, being highest in the Js data set, and lowest in the S II data set (Table S2). Similarly, using the phylogentic distance matrix (see above) sites scores were obtained from the first axis of PCoA both of the active (rBCCp) and total bacterial communities (dBCCp). Communities with similar PCoA site scores have thus closely related non-shared taxa whereas non-shared taxa are more distantly related between communities with different site scores. The first axis explained similar amounts of variation in community composition in all data sets, 19–40% in rBCCp and 22–38% in dBCCp (Table S2), again being highest in the Js data set in both cases.

### Statistical analyses

Average beta-diversity of bacterial communities (OTUs of 99% 16S rRNA similarity) within each data set (rBCCt and dBCCt) was calculated as Morisita-Horn dissimilarities. Morisita-Horn dissimilarities close to zero indicate very similar communnities, whereas values close to 1 indicate completely different communities.

Bray-Curtis dissimilarites of carbon substrate use (AWCD scores of all 31 substrates) were obtained as an estimate of between site variation in trait composition. Bray-Curtis dissimilarities close to zero indicate communities that have a very similar relative use of different carbon substrates, whereas values close to 1 indicate a completely different use of carbon substrates. Non-metric multi-dimensional scaling (nMDS) was performed using PAST version 2.17 [31] to show differences in carbon substrate use and bacterial community composition (BCCt) among data sets.

To study associations between community BPC and local environmental factors, functional trait composition of communities and the different aspects of BCC within data sets, we did partial least square regressions (PLS) with SIMCA 12.0 (Simca 12.0.1., Umetrics AB, Umeå, Sweden). BPC was the dependent variable and TC, TP, TN, TN/TC, TP/TC and average use of the different substrate groups and PCoA sites scores of the first axes of BCC (all BCC measures) were used as explanatory variables (Table 2). PLS has the advantages that it can handle co-variation among variables and is not sensitive to the number of explanatory variables relative sample size as explanatory variables are transformed into one or several latent variables that explain the maximum variance of the dependent variable [32]. From the PLS we extracted Variable Importance for the Projection (VIP-scores) that describe the relative importance of a variable for the correlation between the latent explanatory variables and the dependent variable. VIP-scores>1 indicate important variables, and the higher the value the more important is a variable for the correlation between the latent and dependent variable. We used the scaled and centered coefficients between the explanatory variables and the dependent variable to show the direction of the relationship (positive or negative). To infer covariation between different explanatory variables and BPC we plotted the loadings from the PLS. Explanatory variables with high loadings (positively or negatively) explain most of the variation in the latent variables, and explanatory variables close together show high covariation. In the loading plot, explanatory variables close to BPC are positively correlated with BPC whereas explanatory variables distant from BPC are negatively correlated with BPC. However, to get actual values of correlations between explanatory variables we also calculated Pearson correlation coefficients, *r*. BPC and environmental data was log-transformed prior to the analysis for the data to better fit a normal distribution. We did paired t-tests to compare the explanatory power of active (rBCC) and total (dBCC), respectively phylogenetic based (BCCp) or taxonomic based (BCCt) measures of BCC on BPC.

**Table 2:** Average Bray-Curtis (BC) and Morisita-Horn (MH) dissimilarities.

Data set	BC Biolog	MH rBCCt	MH dBCCt	MH r/dBCCt
Us	0.32 (0.13)	0.82 (0.11)	0.71 (0.06)	0.78 (0.12)
Js	0.22 (0.03)	0.86 (0.17)	0.70 (0.13)	0.84 (0.14)
Uw	0.22 (0.06)	0.71 (0.11)	0.60 (0.12)	0.52 (0.15)
Jw	0.20 (0.08)	0.63 (0.11)	0.45 (0.09)	0.56 (0.07)
S I	0.13 (0.02)	0.71 (0.09)	0.71 (0.09)	0.62 (0.07)
S II	0.13 (0.03)	0.63 (0.07)	0.72 (0.05)	0.70 (0.09)
Average	0.20 (0.06)	0.72 (0.09)	0.66 (0.10)	0.68 (0.12)

Values are calculated between the sampling sites within each data set for carbon substrate use (Biolog, functional trait composition) and BCC, respectively. Standard deviations (SD) are given in parenthesis. MH r/dBCCt is the average of the within sample Morisita-Horn dissimilarity between rBCCt and dBCCt.

To study whether dispersal rates and environmental heterogeneity between communities in a data set may contribute to the relative importance of BCC, functional traits and the environment on productivity we did Pearson correlation between the VIP-scores of explanatory variables in a data set and dispersal level or degree of environmental heterogeneity (CV of environmental variables) in each data set. For functional trait composition and environmental variables VIP values could be used directly. For rBCC (active part) and dBCC (total community) we used the highest VIP-value of BCCt and BCCp, respectively.

## Results and Discussion

In this field study we investigated the potential importance of bacterial community composition (BCC), functional trait composition (Biolog substrate use), and local environment for functioning (bacterial production per cell, BPC) of freshwater bacterial communities. We hypothesized that these relationships may differ depending on environmental heterogeneity and rates of dispersal among communities. Further, we tested the idea that depending on how BCC was determined, the strength of the relationship to functioning would differ. To enable such an evaluation we obtained six different data sets, each consisting of 7–15 freshwater bacterial communities (water and sediments, lakes and streams), in an identical manner, and statistically evaluated the steering factors for functioning.

**Figure 1:**
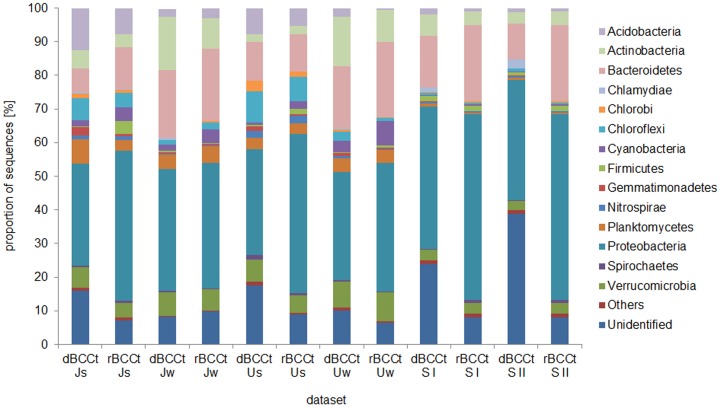
Bar diagrams representing the relative proportion of 16S sequences (rRNA and gene) belonging to the most abundant phyla. ‘Others’ contain OTUs with a relative abundance below 0.5% in the entire data set. ‘Unidentified’ denotes OTUs whose taxonomic affiliation is unknown.

**Figure 2:**
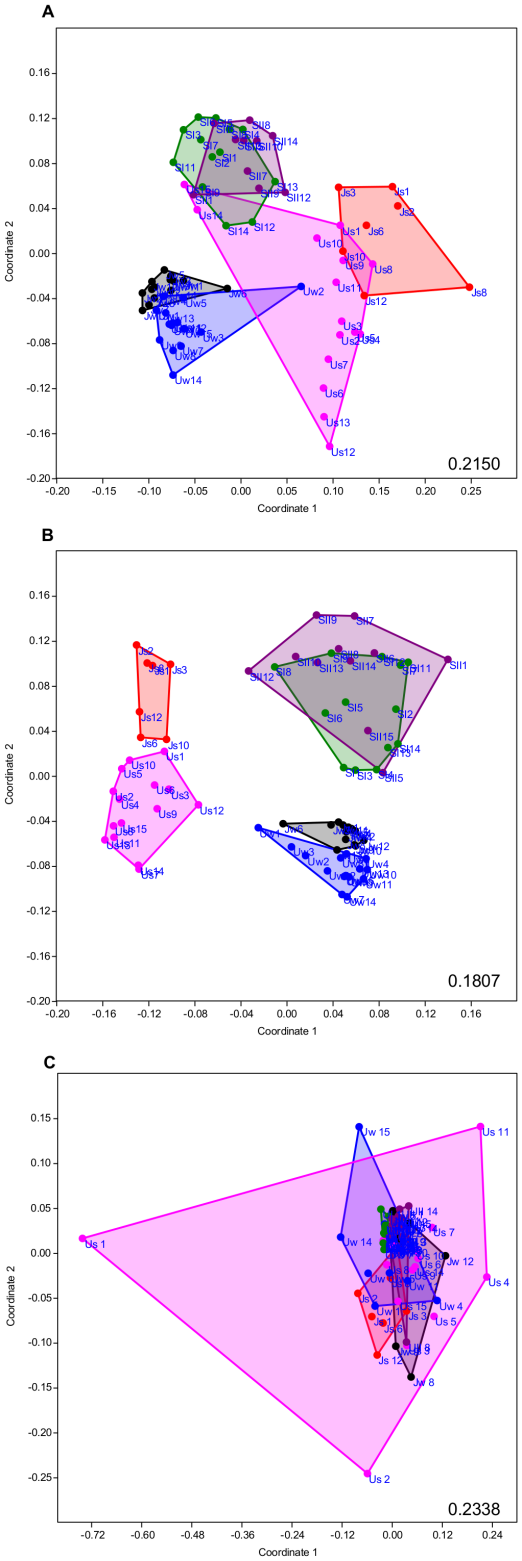
Results from a non-metric multi-dimensional scaling (nMDS) analysis. Depicted are the differences in bacterial community composition between all stations for rBCCt (A) and dBCCt (B), based on Morisita-Horn dissimilarities. The difference in carbon use is based on Bray-Curtis dissimilarities of the Biolog data (C). Stress values are given in the lower right corner.

The bacterial communities were analyzed by 454 sequencing and their compositions were found to be rather typical for freshwaters [33], e.g., dominated by Proteobacteria and Bacteroidetes (Fig. 1) but there were clear differences in bacterial community composition based both on the 16S rRNA and the 16SrRNA gene (Fig. 2A and B).

The average MH dissimilarity among sites was similar for those two BCC measures, i.e. on average 0.72 among all sites and ranging between 0.63 and 0.86 for rBCCt (Table 2, Fig. 2A) and 0.66 on average and ranging between 0.45 and 0.72 for dBCCt (Table 2, Fig 2B). In contrast, the dissimilarity of functional trait composition (Bray Curtis distance of Biolog substrate use) was much lower, the average being only 0.20 (Table 2). The functional trait composition did also not differ among habitat types in an obvious way (Fig. 2C). This may indicate a redundancy of taxa for processing carbon substrates, i.e. that different taxa can perform similarly on the same carbon substrate.

Assuming that rBCCt should reflect the active community and dBCCt the total community [20] the proportion of active taxa appeared to have been low since the average MH dissimilarity between rBCCt and dBCCt of a given community was 0.68 (Table 2). Especially in sediments, many bacterial cells could have been inactive since MH-distances were on average 0.84 and 0.78 for Jämtland and Uppland respectively, while the lake water communities may have shown a greater proportion of active cells, however still showing MH-distances above 0.5 (Table 2). Therefore it may be expected that rBCCt and dBCCt would be differently related to our functional measure BPC. Our hypothesis was that the active community (rBCC) would be better related to functioning than the total community (dBCC). In our analysis we also included two BCC measures based on phylogenetic distances (rBCCp and dBCCp), hypothesizing that a phylogenetic distance measure would show a stronger coupling between BCC and BCP than the rigid OTU definition used to calculate MH dissimilarities. The potential influential factors on BCP were investigated using PLS which is a statistical method suitable for handling many variables with co-variation among them [32].

The latent variable(s) generated from BCC, carbon substrate use and nutrient levels in the PLS explained most variation in BPC in the Jw data set (94%; R2Y in Table 3) and least variation in S I (27%), and more than 50% in all other data sets. Thus, the latent variables explained a major part of variation in BPC in most data sets. In all data sets the PLS identified BCC to explain an important part of the variation in BPC, since one or several measures of BCC had VIP>1, but other factors contributed as well (Table 3). In four of the data sets (both lake water data sets, Js and S I) a measure of BCC had the highest VIP value, i.e. explained most of the variation in BCP (Table 3, Fig. S1). In the remaining two data sets (Us and S II) functional trait composition of communities and the local environmental conditions, respectively, were better explanatory variables. Since the most important factor thus varied among data sets this result highlights the importance of not relying on a single set of data to draw general conclusions regarding drivers of bacterial functioning in nature. In line with these results are several previous studies showing variable relationships between BCC and functioning [6], [7], [8], [9], [34], [35].

**Table 3:** VIP values from PLS analysis between BPC and the explanatory variables for the data sets.

			Data set			
Model fit	Us	Js	Uw	Jw	S I	S II
R2X	23	23	28	56	26	25
R2Y	46	79	54	94	27	51
**Explanatory variable**						
rBCCt	**1.11 (−)**	0.46 (+)	**1.88 (+)**	**1.76 (−)**	**1.50 (+)**	**1.38 (−)**
rBCCp	**1.02 (−)**	0.36 (+)	0.31 (+)	0.75 (−)	0.11 (−)	0.60 (+)
dBCCt	0.28 (+)	0.56 (+)	0.19 (−)	0.51 (+)	**1.73 (−)**	0.25 (−)
dBCCp	0.95 (−)	**2.25 (−)**	0.45 (−)	0.73 (−)	**2.06 (+)**	1.13 (−)
amino acids	0.47 (+)	**1.28 (+)**	0.56 (−)	0.86 (−)	**1.18 (−)**	0.38 (−)
aromatic	0.43 (−)	**1.21 (−)**	**1.11 (+)**	**1.10 (+)**	**1.24 (+)**	0.94 (−)
simple	**1.13 (−)**	**1.31 (−)**	0.4 (−)	**1.06 (−)**	0.26 (−)	0.28 (+)
polymer	0.33 (+)	0.15 (+)	0.5 (+)	0.63 (−)	0.09 (−)	0.31 (−)
cyclic	**1.92 (+)**	**1.53 (+)**	0.33 (−)	0.62 (+)	0.06 (−)	1.21 (+)
TN	1.10 (−)	0.43 (−)	**1.22 (+)**	0.87 (+)	0.22 (−)	1.51 (−)
TN/TC	0.89 (+)	0.09 (+)	0.67 (+)	**1.25 (+)**	0.80 (+)	0.53 (+)
TC	**1.28 (−)**	0.04 (−)	0.1 (+)	0.64 (−)	0.41 (−)	**1.56 (−)**
TP	0.61 (−)	0.24 (+)	**1.83 (+)**	**1.22 (+)**	0.30 (+)	**1.54 (−)**
TP/TC	**1.13 (+)**	0.92 (+)	**1.65 (+)**	**1.20 (+)**	0.73 (+)	0.58 (−)

VIP>1, identifying variables most relevant for explaining BPC, are shown in bold. The direction of the association between BPC and the explanatory variables is deduced from scaled and centered coefficients (CoeffCS) and given in parenthesis. R2X is the proportion of variation in the explanatory data set explained by the latent factor(s), and R2Y is the proportion of variation in BPC explained by the latent factor(s) from the explanatory data set.

A methodological consideration arising from these data is that if we had used only one BCC measure instead of four to infer the general importance of BCC for functioning we would have perceived it to be smaller, since “best” BCC measure varied among data sets. However, contrary to our expectations we did not find rBCC (active community) to be more closely coupled to functioning than dBCC (total community) because rBCC did not have higher VIP-values than dBCC (paired t-test: t_10_ = 0.3, p = 0.8). Further, phylogenetic measures of beta-diversity (BCCp) did not explain more variation than those based on the distance of OTUs (BCCt) (paired t-test: t_(10)_  = 0.1, P = 0.9). In fact, the BCC measure taking into account the phylogenetic relatedness of the active compartment of the community (rBCCp) performed “worst” with only oneVIP-value>1 (Uppland sediment VIP = 1.02). Methodological reasons for these unexpected results may be that rRNA based methods are poor measures of actual activity [36], for instance overestimating the proportion of active cells [37]. Thus, it is unclear what exactly our rBCC measure represents. Moreover, there are limitations in using phylogeny-based estimates of BCC for communities for which the evolutionary history of its constituents is unknown [38]. A problem when assessing phylogenetic diversity of bacterial communities can be that the large number of taxa requires a huge number of variable sites in the gene or genome to get accurate phylogenetic trees to obtain a well-represented estimate of phylogenetic similarity between communities. In our case we could only estimate phylogenetic similarity from less than 25% of total numbers of read (including more taxa would have generated uninformative phylogenetic trees) so our estimates may differ from ‘true’ phylogenetic similarities between communities. Moreover, the phylogenetic distance estimate we used (PCD) does not account for differences in relative abundance which may be important for community functioning. Thus, we can conclude that methods development is a necessity, both when it comes to accurately define the active proportion of the community, as well as to how phylogenetic diversity should be determined.

At least one of the functional trait groups (i.e. the ability to use certain organic compounds in Biolog plates) showed VIP>1 in each data set (Table 3, Fig. S1). The strongest positive association to any of the functional trait groups was either to aromatic compounds or cyclic compounds (at least three cases with VIP>1 each). This is in line with a previous experimental study where the communities' potential to use aromatic compounds was a key functional trait contributing to variation in bacterial functioning [12]. In contrast, polymers and simple substrates never showed a strong positive association to BPC. Functional traits with high VIP were relatively weakly correlated with BCC measures in both the Uppland and both stream data sets (r<0.31), but showed considerably stronger correlations in the two Jämtland data sets (r>0.45, Table 4). Weak correlations between functional traits important for BPC (high VIP) and BCC in addition to a large variation in BCC among sites compared to the variation in functional traits strongly imply a functional redundancy of the different bacterial communities. However, in most cases BCC explained more variation in productivity than trait composition. This indicates that there are other taxa specific traits than those measured in Biolog plates that also contribute to productivity, e.g., the use of other resources or a very specific resource. Considering that the aquatic dissolved organic carbon pool is complex and poorly characterized [39] it is not an easy task to pinpoint which organic carbon processing traits should be of interest for a mechanistic understanding of bacterial growth, but should be a subject of future research.

**Table 4:** Pearson correlation coefficients between BCC measures (in parenthesis) with VIP-values>1 from the PLS.

Data set	Amino acid	Aromatic	Simple	Cyclic	TC	TN	TP	TN/TC	TP/TC
Us (rBCCt)			−0.31	−0.22	−0.32				
Js (dBCCp)	−0.33	0.46	0.75	−0.69					
Uw (rBCCt)		0.30				0.38	0.63		0.74
Jw (rBCCt)		−0.45	0.41			−0.60		−0.67	−0.62
S I (dBCCp)	−0.16	0.18							
S II (rBCCt)				−0.05	0.20	0.40	0.33		

BPC in the second stream sampling was best explained by a negative association with TC, TP, and TN (Table 3, Fig. S1). These changes in the environment were not evidently strongly associated with changes in BCC (Table 4). Also in both the lake water data sets BPC was positively correlated with nutrient levels or ratios between different nutrients and rBCC was well correlated with these environmental changes (Table 4). The influence of local environmental variables on bacterial production was strongly reduced by using BPC, and not gross community production as a response variable in the PLS (results not shown). However, local environmental conditions still showed associations with productivity in all data sets except two (Js and S I). Since a limitation of BPC by C or P alone or the combination of C and P or N is often found in Swedish lakes [40], [41], it is not surprising that it was the inorganic nutrient availability and its relation to carbon that explained most variation in productivity. This would indicate that the stoichiometry of nutrients is more important for BPC than nutrient levels per se. In the lake water data sets there was a relatively high covariation between environmental variables and BCC variables contributing to variation in BPC, indicating that the environment partly drives changes in BCC that affect BPC. The negative relation between productivity and nutrient levels in the second stream sampling was surprising and could be a case of negative density dependent interactions, e.g., other nutrients (not considered here) become constraining or there are antagonistic interactions between cells.

**Figure 3:**
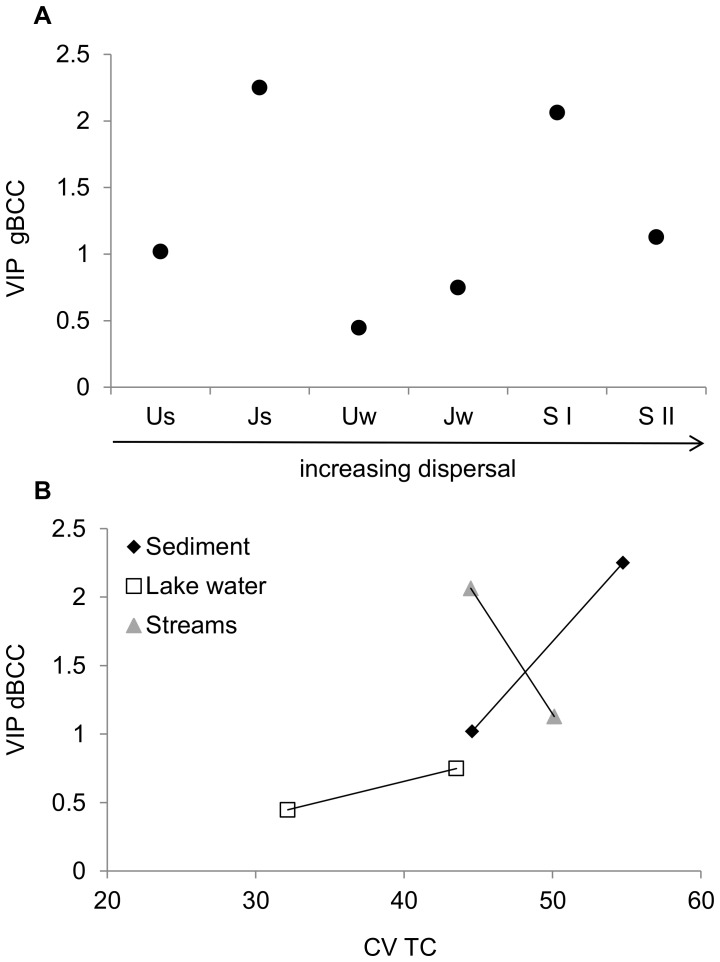
VIP-values of dBCC measures on BPC in relation to dispersal rate (A) and environmental heterogeneity (B). Environmental heterogeneity was measured as coefficient of variation (CV) of total carbon (TC).

Since the strength of the association between BCC and BPC differed between data sets, it seems to be context dependent. A previous modeling study linking diversity and functioning suggested this strength to depend on environmental heterogeneity and species specific traits in relation to the environment [42]. We hypothesized that the dispersal rate among communities would determine the degree of species sorting and in turn the strength of the coupling between BCC and functioning. However, the explanatory power of BCC (rBCC or dBCC) for BPC (VIP-scores) showed no consistent association with the anticipated dispersal rates among the datasets (Fig. 3A). There was a stronger coupling between BCC and BPC in data sets with high environmental heterogeneity in organic carbon but it was not significant (r = 0.79; P = 0.06; Fig. 3B), maybe partly due to too low sample size. Thus, we could not show a link between different community assembly processes and strength of BCC-functioning relationships. One explanation to this result could be that the range in relative importance of different community assembly processes was not very large in our study. For instance none of the communities were truly isolated and, thus, probably did not experience dispersal limitation. It can also be questioned if dispersal rates were high enough to cause mass effects [10]. Thus, the communities investigated here may all have been formed by local species sorting processes to a similar extent. Future studies aiming to explore the effect of community assembly for the connection between community composition and functioning may, thus, aim for greater gradients in dispersal as well as environmental heterogeneity, also including more disturbed environments than we did, thereby including communities being more likely assembled in different ways. For instance isolated sites such as ground water pockets and environmental gradients of greater range and intensity, such as low pH and high salinity could be included.

In summary, BCC was associated with per cell productivity in all six data sets, but which measure of BCC was best associated differed between data sets. We could not find any consistent difference in BCC-functioning relationship using phylogenetic or non-phylogenetic measures of BCC, nor using BCC from active bacteria or the total bacterial community, highlighting the problem of choosing the best method for BCC measurements in such highly diverse communities. Neither could we find any clear pattern in the relative importance of BCC for functioning between data sets. Thus, the coupling between BCC and functioning in aquatic bacteria seemed context-dependent, but here we could not dissect what the context was. We therefore expect that future research in the area will require methodological considerations on how to measure the active compartment of communities as well as which beta-diversity measures to use. The importance of functional carbon processing traits for productivity was to some extent supported by the compounds tested here, suggesting an importance of the capacity to use especially aromatic and cyclic compounds. However, the results also suggest that important traits still remain to be identified.

## Supporting Information

Figure S1 Loading (w*c) bi-plots (of the first and second latent factor) between explanatory variables and per cell bacterial productivity (BPC, filled square) from the PLS for each data set. Explanatory (X) variables are split into functional traits of the community (open circle), environment conditions (open triangle) BCC (cross). Us =  Uppland sediment, Js =  Jämtland sediment, Uw =  Uppland lake waters, Jw =  Jämtland lake waters, S I =  First stream sampling, and S II =  Second stream sampling. For Us, Js and Uw the loadings at the second axis are only shown for illustration purposes as only one latent variable was calculated.
(TIF)

Table S1 **Overview of abbreviations used throughout the article.**
(DOCX)

Table S2 **Eigenvalues and variation explained by the first Principal Coordinate Axis (PcoA) of the different BCC for each data set.**
(DOCX)

Data S1 **Raw data.**
(XLSX)
