# Supplementary figures and images for: Relationships between Bacterial Community Composition, Functional Trait Composition and Functioning Are Context Dependent – but What Is the Context?
Source: PLoS One. 2014 Nov 7;9(11):e112409. doi: 10.1371/journal.pone.0112409 (PMC4224428; doi:10.1371/journal.pone.0112409)

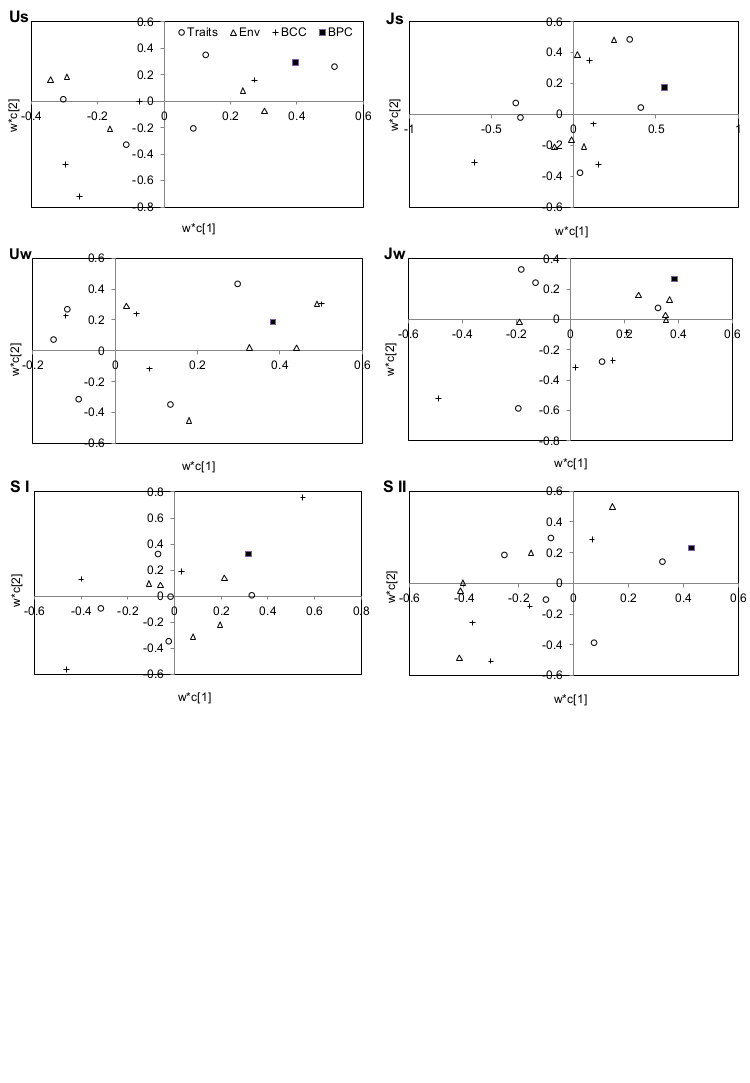

Supplement: Figure S1 — Loading (w*c) bi-plots (of the first and second latent factor) between explanatory variables and per cell bacterial productivity (BPC, filled square) from the PLS for each data set. Explanatory (X) variables are split into functional traits of the community (open circle), environment conditions (open triangle) BCC (cross). Us = Uppland sediment, Js = Jämtland sediment, Uw = Uppland lake waters, Jw = Jämtland lake waters, S I = First stream sampling, and S II = Second stream sampling. For Us, Js and Uw the loadings at the second axis are only shown for illustration purposes as only one latent variable was calculated. (TIF) [file pone.0112409.s001.tif]
