# Supplementary material for: Relationships between Bacterial Community Composition, Functional Trait Composition and Functioning Are Context Dependent – but What Is the Context?
Source: PLoS One. 2014 Nov 7;9(11):e112409. doi: 10.1371/journal.pone.0112409 (PMC4224428; doi:10.1371/journal.pone.0112409)
Supplement: Table S1 — Overview of abbreviations used throughout the article. (DOCX) [file pone.0112409.s002.docx]

**Table S1: Overview of abbreviations used throughout the article.**

| BCC | bacterial community composition |
| --- | --- |
| BCCt | taxonomic component of bacterial community composition |
| BCCp | phylogenetic component of bacterial community composition |
| rBCC | 16R rRNA-based bacterial community composition ('active community') |
| dBCC | 16S rRNA gene-based bacterial community composition ('total community') |
| rBCCt | taxonomic component of the 16S rRNA-based bacterial community composition |
| dBCCt | taxonomic component of the 16S rRNA gene-based bacterial community composition |
| rBCCp | phylogenetic component of the 16S rRNA-based bacterial community composition |
| dBCCp | phylogenetic component of the 16S rRNA gene-based bacterial community composition |
| OTU | operational taxonomic unit |
| BPC | bacterial per cell productivity |
| TC | total (non-purgeable) carbon |
| TN | total nitrogen |
| TP | total phosphorus |
| TN/TC | total nitrogen normalized to total carbon |
| TP/TC | total phosphorus normalized to total carbon |
| AWCD | average well color development |
| CV | coefficient of variation |
| PLS | partial least square regression |
| VIP | variable importance for the projection |
| MH | Morisita-Horn |
| BC | Bray-Curtis |
| PCoA | principal coordinate analysis |
| R2X | variation explained by explanatory data by the PLS model |
| R2Y | variation explained of dependent variable by the PLS model |
| Js | Jämtland sediment data set |
| Jw | Jämtland lake water data set |
| Us | Uppland sediment data set |
| Uw | Uppland lake water data set |
| S I | 1^st^ stream sampling data set |
| S II | 2^nd^ stream sampling data set |
