# Supplementary material for: Relationships between Bacterial Community Composition, Functional Trait Composition and Functioning Are Context Dependent – but What Is the Context?
Source: PLoS One. 2014 Nov 7;9(11):e112409. doi: 10.1371/journal.pone.0112409 (PMC4224428; doi:10.1371/journal.pone.0112409)
Supplement: Table S2 — Eigenvalues and variation explained by the first Principal Coordinate Axis (PcoA) of the different BCC for each data set. (DOCX) [file pone.0112409.s003.docx]

**Table S2**: **Eigenvalues and variation explained by the first Principal Coordinate Axis (PcoA) of the different BCC for each data set.**

| **Data set** | **rBCCt** | **dBCCt** | **rBCCp** | **dBCCp** |
| --- | --- | --- | --- | --- |
| Js | λ=0.8, %=42 | λ=0.7, %=70 | λ=1.3, %=40 | λ=1.8, %=38 |
| Jw | λ=0.5, %=16 | λ=1.6, %=50 | λ=2.3, %=24 | λ=1.6, %=26 |
| Us | λ=0.8, %=21 | λ=1.6, %=39 | λ=2.1, %=27 | λ=2.4, %=22 |
| Uw | λ=0.6, %=14 | λ=1.5, %=41 | λ=1.6, %=19 | λ=2.9, %=28 |
| S I | λ=0.6, %=16 | λ=1.4, %=46 | λ=1.3, %=31 | λ=1.4, %=27 |
| S II | λ=0.5, %=12 | λ=0.9, %=33 | λ=1.7, %=19 | λ=1.4, %=28 |
